# Broncho Pneumonia and Abscess of the Lung

**Published:** 1906-06-02

**Authors:** 


### BRONCHO-PNEUMONIA AND ABSCESS OF THE LUNG.

In the current issue of the *Glasgow Medical Journal* Mr. Ivy M'Kenzie has an instructive paper upon this subject. He notes the wide diversity of opinion which is to be met with as regards the meaning to be attached to the term broncho-pneumonia, a diversity due to the great variety of pathological appearances which are associated with this disease as commonly understood. The paper in question is based upon observations of broncho-pneumonia following measles, whooping-cough, and diphtheria. In the catalogue are 1,000 cases of measles, and 130 post-mortem examinations of broncho-pneumonia secondary to the three specific fevers above mentioned. The following three varieties of abscess were met with in these examinations: (*a*) Small punctiform collections of pus. When these occur they are numerous and affect those portions of the lung in which the inflamatory changes are most advanced; they appear to originate in the central softening of a consolidated pulmonary lobule. Microscopical examination of such a lung shows dense masses of pus-cells in the centre of the lobule, while the peripheral alveoli, though collapsed and infiltrated, remain, nevertheless, easily recognisable. This condition is suppurating broncho-pneumonia. (*b*) The second variety consists of abscesses of larger size and less abundance. This variety differs from the first only in degree and does not admit of any clinical differentiation. (*c*) The third variety is marked, in the author's opinion, by the late appearance of the abscess, at a period, that is to say, after the disappearance of the broncho-pneumonia proper. One case of this variety is described in detail. The patient was a delicate child of 1½ years who developed measles, and then consecutively bronchitis and broncho-pneumonia. The latter lasted a month, and passed to a favourable termination. After a fortnight's afebrile convalescence there began an intermittent fever, without any notable pulmonary embarrassment or constitutional disturbance. The physical signs were suggestive of empyema or abscess at the right apex, but exploratory puncture was negative. The fever ended suddenly at the end of three weeks, and was followed by a second period of convalescence lasting eighteen days, during which the patient made rapid progress toward recovery. At the end of this period of convalescence the patient again became suddenly and seriously ill, with a remittent fever, great prostration and weakness, attacks of vomiting and cyanosis. Death occurred on the fifth day of this third attack, the only marked alteration in the physical signs consisting in an extension of the cardiac dulness, with some indistinctness of the heart-sounds. Post-mortem the pericardial cavity was found to be distended by half-a-pint of sero-purulent fluid in which were suspended particles of fibrin. The left pleural cavity contained a considerable effusion, described as clear and serous. The right lung was crepitant throughout except at the right apex, where was a patch of consolidation with a cavity at its centre about the size of a walnut. This cavity was empty and smooth. The left lung was normal throughout. Films from the pericardial fluid contained capsulated diplococci, as did similar preparations of the spleen-pulp. Culturally, the pericardial fluid yielded a pure culture of the pneumococcus.

The pathological history of this case is reconstructed by the author as follows: An original broncho-pneumonia in a delicate rickety child and one for this reason predisposed to pulmonary atelectasis: a patch of atelectasis with inflammation and pleurisy occurs at the right apex; the chest weakness, the situation of the lesion and the pleuritic adhesions all tend to delay resolution; the functionless area becomes the seat of a secondary pneumococcal infection which leads ultimately to abscess formation. Attention is drawn to the interesting sidelight on immunity which is offered by this case. The pneumococcus was here responsible for an illness extending over twelve weeks, with two intervals of two and of three weeks respectively. It is to be assumed that a temporary immunity, leading to recovery and two weeks of convalescence was followed by a re-infection, in the course of which the organism took on a pyogenic character. This condition lasted three weeks and was again followed by a period of immunity lasting almost three weeks, at the end of which time the organism again became active and gave rise to a fatal septicæmia.

The author gives a summary of views as to the intimate pathological processes associated with broncho-pneumonia in general. These changes are described by V. Ziemssen as follows: The disease begins as an intense catarrhal inflammation of the smallest bronchi; then follow atelectatic changes in the lobules of lung supplied by the affected tube, the lumen of which becomes more or less completely occluded by inflammatory products. The subsequent nutritional and inflammatory changes in the collapsed lobule constitute lobular pneumonia. As regards the factors which determine the site of the atelectasis, the author states that the sites of election are these: (1) The posterior borders of the lower, and sometimes also of the upper, lobes; (2) the narrow wedge-shaped extremities lying between the dome of the diaphragm and the chest wall; (3) the lingula of the left lung. It is pointed out that in a patient lying supine and prostrate from fever, catarrhal secretions tend to gravitate to the posterior and less active portions of the lung while cough is often diminished by the severity of the intoxication, a fact which, associated with the weakness of the chest-wall in early life and the frequently debilitated state of the patient, is quite sufficient to prevent the inflation of collapsed lung.

